# Titanium Coated with Graphene and Niobium Pentoxide for Biomaterial Applications

**DOI:** 10.1155/2022/2786101

**Published:** 2022-11-30

**Authors:** Hazel Paloma Reis Corado, Francielly Moura de Souza Soraes, Dyanni Manhães Barbosa, Andreza Menezes Lima, Carlos Nelson Elias

**Affiliations:** Instituto Militar de Engenharia—IME, Department of Materials Science, Praça General Tibúrcio, 80, Praia Vermelha, Urca, CEP 22290-270, Rio de Janeiro, RJ, Brazil

## Abstract

Graphene and niobium oxide are used in biomaterial coatings. In this work, commercially pure titanium (cp Ti) was coated with graphene oxide (GO), niobium pentoxide (Nb_2_O_5_), and a mixture of both materials (NbGO) by the electrochemical deposition method. The surface morphology, roughness, wettability, and degradation of coated and uncoated samples were analyzed by scanning electron microscopy, interferometry, and contact angle. The results showed that the specimens coated with NbGO (cp Ti-NbGO) showed the highest surface roughness (Ra = 0.64 *μ*m) and were hydrophobic. The contact (*θ*) angle between water and the surface of uncoated specimens (cp Ti), coated with GO (cp Ti-GO), coated with a mixture with GO and Nb_2_O_5_) (cp Ti-NbGO), and coated with Nb_2_O_5_ were 50.74°, 44.35°, 55.86°, and 100.35°, respectively. The electrochemical corrosion tests showed that coating with graphene oxide increased the corrosion resistance and coating with Nb_2_O_5_ decreased the corrosion resistance. The negative effect of the effect of Nb_2_O_5_ coating in corrosion resistance compensated for the release of Nb_2_O_5_, which helps osseointegration, increasing cell viability, and proliferation of osteoblasts. The NbGO coating may be a good way to combine the bactericidal effect of graphene oxide with the osseointegration effect of Nb_2_O_5_.

## 1. Introduction

Titanium and its alloys are widely used as biomaterials. Its main feature is osseointegration since the titanium oxide film on the surface enhances the adhesion of proteins and bone cells. The wrought titanium 6-aluminum4-vanadium ELI alloy (Ti-6Al-4V ASTM F136), alpha plus beta titanium alloy forgings (ASTM F620), and wrought titanium 6-aluminum7-niobium alloy (ASTM F1295) have adequate mechanical properties for use as a biomaterial, but they release toxic ions (Al and V), decreasing its biological activity. Another problem observed is that among the different causes of failures of orthopedic prostheses and dental implants, the adhesion of biofilms and chronic inflammatory processes deserves to be highlighted. In the case of implants, biofilm adhesion can also facilitate peri-implantitis. Despite the various procedures adopted to minimize this problem, implant scraping and the use of antibiotics are the most efficient. Even so, the inflammatory process can be recurrent, leading to implant loss. For this reason, surface treatment and chemical modifications of titanium alloys for implant biomaterials application are important.

Graphene oxide (GO) has a bactericidal effect and does not inhibit cell growth. The antibacterial activity of GO is based on the production of hydroxyl radicals, which attack the cell membranes of bacteria, causing their death. During the process, reactive oxygen species are generated, which lead to oxidative stress, exceeding the antioxidant defense capacity of the bacteria and causing damage to their lipids, proteins, and DNA. The morphology of GO is formed by nanosheets with sharpened edges that break the bacterial membrane, causing the intracellular matrix to drain into the medium [1]. GO inhibits the growth of bacteria such as *Escherichia coli* and *Streptococcus iniae*. In high concentrations, GO inhibits the action of Gram-negative bacteria [2].

Among niobium oxides, niobium pentoxide (Nb_2_O_5_) is the most stable thermodynamically [3]. Cell culture results showed that coating with Nb_2_O_5_ enhances cellular viability and proliferation after 21 days. The cells present good adhesion and are homogeneously distributed on the surface. Previous results showed that the lactate dehydrogenase activity was similar to the titanium surface with and without Nb_2_O_5_ coating, indicating a similar number of cells.

It is extremely important to evaluate metallic implant corrosion resistance to avoid degradation and release of toxic ions. The accelerated degradation causes problems in tissue, but in some cases, it can be beneficial [4]. Literature results report the corrosion of dental implants made with titanium alloy ASTM F136 in contact with saliva and fluoride, which can cause the release of ions that can induce toxicity in the organism and loosen the implant [4–6]. Coating titanium with GO may increase the corrosion resistance of dental implants.

This work aims to examine the properties of cp Ti coated with graphene oxide, niobium pentoxide, and the mixture of both materials for use in dental implants.

## 2. Materials and Methods

Commercially pure titanium grade 4 bars (cp Ti ASTM G4) were used in the present work. The material was supplied by Conexão Sistema e Prótese (Arujá, SP-Brazil). The samples were acid-etched with a mixture of sulfuric acid, hydrochloric acid, and distilled water and washed in an ultrasonic bath with distilled water before all testing.

**Figure 1:**
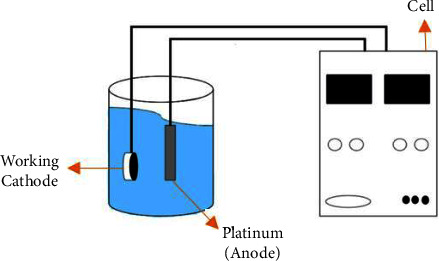
Schematic drawing of the electrochemical deposition cell. The working cathode was the analyzed specimen.

The deposition of graphene oxide was carried out by electrodeposition in an aqueous solution (Figure 1). The graphene oxide concentration was between 0.5 mg/mL and 0.7 mg/mL. During deposition, the working electrode (cathode) was a cp Ti sample, and the anode was a platinum plate. The distance between the cathode and the anode was 1 cm. The depositions of Nb_2_O_5_ and a mixture of GO and deposition and Nb_2_O_5_ followed similar routes.  Table 1 shows the code used to identify the specimens.

## 3. Characterization

The surface morphology of three samples from each group was investigated by scanning electron microscopy (Field Emission Gun FEI Quanta FEG 250, Hilesboro, Oregon USA) before and after coating.

The sample wettability was measured by the contact angle method. Contact angle measurements were performed with deionized water, using the sessile drop technique. The equipment used was the FTA 100 goniometer (First Ten Angstroms, Portsmouth, VG, USA).

Literature results showed that osseointegration is the main parameter for the success of dental implants, which means a protein and bone cell attachment on the implant surface [7]. This attachment is influenced by the implant surface roughness. Among the parameters that characterize the surface roughness, the arithmetic average roughness (Ra), the average absolute value of the five highest peaks and the five lowest valleys over the evaluation length (Rz), and the root mean square roughness (RMS) were measured before and after the surface treatments. The roughness measurements were performed using Zygo NewView 7100 optical interferometry (Zygo Corporation, Connecticut, the United States).

Two electrochemical analyses were carried out to evaluate the adhesion of the coating on sample surfaces and to analyze the influence on corrosion resistance. The first electrochemical testing was the evolution of the open circuit potential (OCP), which was monitored for 3600 seconds. The second performed testing was potentiodynamic polarization to obtain the polarization curves. For potentiodynamic polarization, a sweep rate of 0.001 V/s and potentials of −0.500 V to 1 V were used. The electrolyte was a physiological solution containing 0.9% of NaCl at room temperature. The equipment used was an AUTOLAB potentiostat/galvanostat (Metrohm Autolab B.V., 3526 KM Utrecht, The Netherlands). The cell used for the analyses consists of a calomel reference electrode, a platinum counter electrode, and the sample disc as the working electrode.

The statistical analyses were performed by an ANOVA variance calculation followed by Tukey's test. For statistical analysis, PRISM GRAPH 9 (Graphstats Technologies Private Limited, Karnataka, India) software was used.

## 4. Results

**Figure 2:**
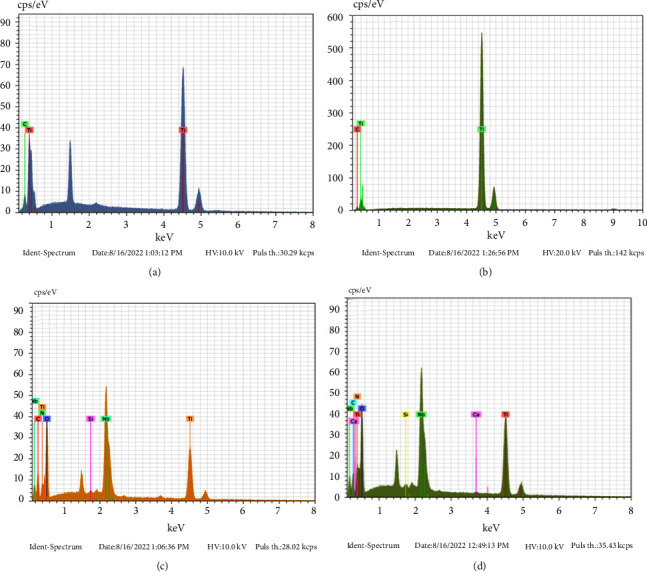
EDS spectra of chemical composition analysis of Ti surface before and after graphene oxide and niobium pentoxide coating. (a) cp Ti before coating. (b) cp Ti-GO. (c) cp Ti-NB. (d) cp Ti-NBGO.

Figure 2 shows the spectra obtained in the semiquantitative chemical analysis with EDS. The surface composition did not change after graphene oxide deposition (Figure 2(b)).  Figure 2(c) shows the EDS spectrum after niobium pentoxide deposition, and it is possible to observe the presence of Nb. After graphene and niobium pentoxide deposition on the surface, the EDS spectrum shows the presence of Nb, as well as C and Ti.

**Figure 3:**
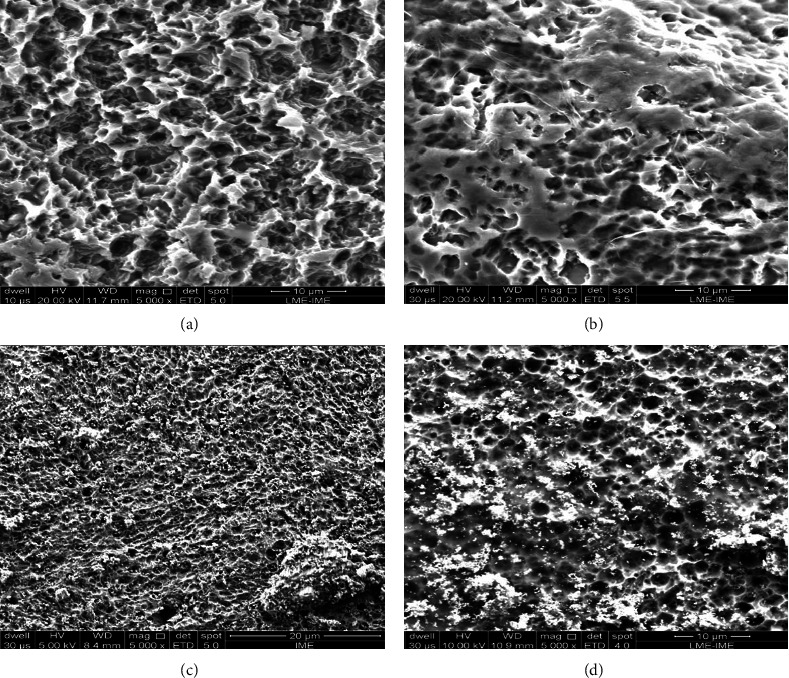
Ti surface morphology before and after graphene coating at low magnification. (a) cp Ti before coating. (b) cp Ti-GO. (c) cp Ti-NB. (d) cp Ti-NBGO.

**Figure 4:**
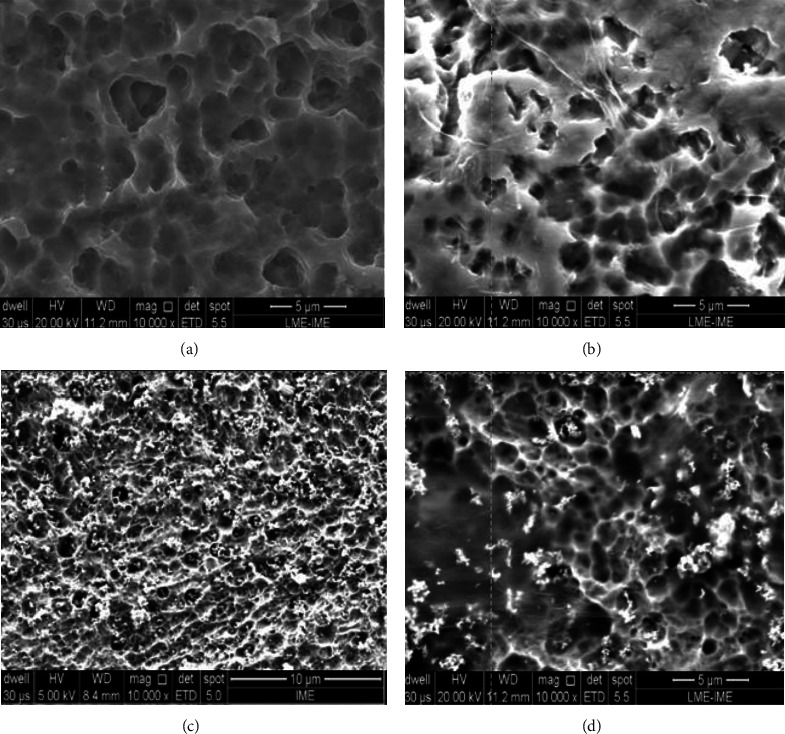
Ti surface morphology before and after graphene coating. (a) cp Ti before coating. (b) cp Ti-GO. (c) cp Ti-NB. (d) cp Ti-NBGO.

Figures 3(b) and 4 show the surface morphology of the samples before and after graphene and niobium oxide coating. Figures 3(b) and 4(a) show that acid-etched cp Ti has a homogeneous surface with microcavities. This morphology is very similar to that observed in dental implant surfaces [8] and leads to good surface protein interaction, cell adhesion, implant osseointegration, and a high dental implant success rate (95–98%). Figures 3(b) and 4(b) show the Ti surface coated with GO; it is possible to observe a film of graphene sheets. Figures 3(b) and 4(c) show some circular Nb_2_O_5_ particles. Figures 3(b) and 4(d) show some Nb_2_O_5_ particles and translucent graphene sheets.

**Figure 5:**
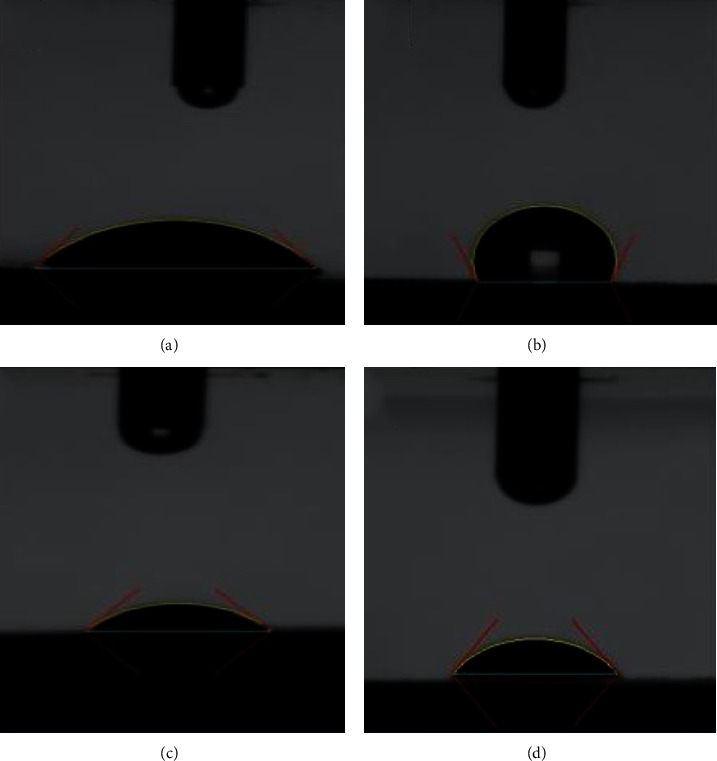
Water drops on the surface. (a) cp Ti-GO (hydrophilic). (b) cp Ti-NB (hydrophobic). (c) cp Ti-NBGO (hydrophilic). (d) cp Ti hydrophilic.

Figure 5 shows water droplets on titanium surfaces. The surface properties influence protein adhesion and cell proliferation. In the initial phase of contact of the biomaterial with the organism, there are physical interactions, chemical bonds between cells and surfaces, or, indirectly, through a change in the adsorption of conditioning molecules, proteins, for example [9]. Cell attachment and expansion are lower on hydrophobic surfaces than those on hydrophilic ones. Moderately hydrophilic surfaces promote cell attachment [10]. Ti with a hydrophilic surface (contact angle <90°) has a lower time for osseointegration than Ti with a hydrophobic surface (contact angle >90°) [11]. The wettability results of all analyzed samples are adequate based on data reported in the literature.

**Figure 6:**
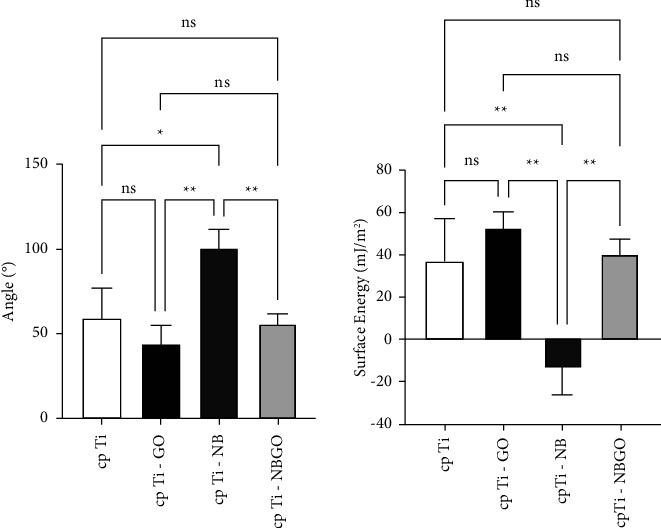
Tukey test results for wettability data.

Table 2 shows that the cp Ti-Nb specimens have a contact angle of 100.35°, which is typical of hydrophobic behavior. Three groups of specimens (cp Ti, cp Ti-GO, and cp Ti-NBGO) have a contact angle lower than 90°, which is hydrophilic behavior.  Figure 6 shows the comparative analysis of the Tukey test of wettability data. It can be seen that there is no statistically significant difference among the hydrophilic groups, and there is a significant difference between the hydrophilic groups and the Ti-NB group.

Roughness and wettability are important parameters to evaluate the success of dental implants. Hydrophobic surfaces decrease the primary interactions with cells in an aqueous environment. It is usually reported that biomaterial surfaces with moderate hydrophilicity improve cell growth and material biocompatibility [10].

Graphene oxide, obtained by the oxidation of graphite flakes, has hydrophilic characteristics, good dispersion, and compatibility with various polymeric matrices [12, 13] Titanium, by itself, is normally hydrophilic. However, most available commercial implants after surface treatment are hydrophobic [14].

The analysis of the simultaneous influence of surface wettability, roughness, and chemical composition on osseointegration is complex. Surface treatments modify the wettability of implants, making them hydrophilic or hydrophobic. Some proteins interact with hydrophilic surfaces, and others interact with hydrophobic ones. Smooth surfaces are suitable for interaction with fibroblasts, and rougher surfaces are suitable for interaction with osteoblasts. As for the wettability, it is possible to estimate the relationship between the surface energy and the contact angle. There is an inversely proportional relationship between the two properties, regardless of whether the samples have micrometer (Ti G4) or submicrometer (Ti Hard) grain size [15].

Cell adhesion and scattering occur on surfaces with moderate wettability and contact angles between 50° and 80°. The fixation and proliferation of osteoblasts increase as the surface wettability increases [16, 17]. The implant osseointegration rate (BIC: bone implant contact) with commercially SLActive surface (hydrophilic, 48.3%) is higher than for implants with hydrophobic surfaces of SLA implants, 32.4% [18]. Cell adhesion and propagation occur on surfaces with moderate wettability and contact angles of 50°–80°.

Classifying surfaces as hydrophilic or hydrophobic is not enough to determine a good relationship between the material surface properties and cellular adhesion and to analyze the possibility of implant osseointegration [19]. The influence of wettability must take roughness into account since there is a correlation between roughness and wettability. The contact angle increases as roughness parameters (Ra, PV, and RMS) increase in a nonlinear way [15].

**Figure 7:**
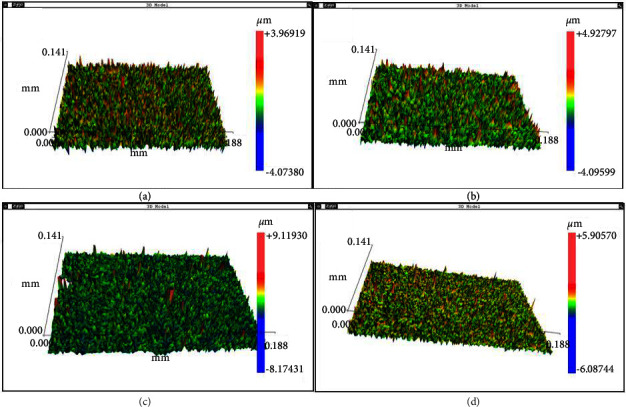
Surface morphology of specimens. (a) cp Ti. (b) cp Ti-GO. (c) cp Ti-NB. (d) cp Ti-NBGO. The color scale on the right side indicates the size of surface irregularities in the form of peaks and valleys. The green color indicates the presence of surface cavities, and the red color indicates the peaks.

Figure 7 shows the surface morphology of the specimens determined by interferometry, and Table 3 shows the surface roughness parameters of the samples analyzed in this study. The specimens had Ra roughness values similar to those reported in the literature [20]. For uncoated samples, Ra = 0.52 ± 0.06 *μ*m. Samples coated with niobium pentoxide had Ra 0.44 ± 0.03 *μ*m. The samples coated with graphene oxide had Ra = 0.47 ± 0.04 *μ*m. Statistical analysis showed no statistically significant difference among three of the four groups studied. The cp Ti-NBGO group had the highest average roughness (Ra = 0.64 ± 0.47 *μ*m), which represents a statistically significant difference from the other groups.

The Ra parameter is most used in the literature to characterize the surface roughness of dental materials and applies to most manufacturing processes. Among the disadvantages of using the Ra value, we highlight the fact that this parameter only quantifies the average roughness. It makes no distinction between peaks and valleys and does not define the shape of the irregularities. For this reason, in the present work, the roughness measurements were complemented by the RMS and Rz parameters. The RMS value corresponds to the square root of the mean of the squares of the ordinates, that is, the root means square deviation. The Rz parameter corresponds to the arithmetic mean of five values of the distances between peaks and valleys. These parameters were quantified to analyze the influence of coatings on surface roughness, which act directly on the cell's adsorption of micrometric surface characteristics [21].

The cell interaction with the implant surface is influenced by topography at macroscopic levels and roughness at the microscopic level. Increasing the surface area of the implant increases the number of possible sites for cell attachment, facilitates tissue growth, and increases mechanical stability. However, this is not a general rule. The level of roughness must be controlled because the cells need anchorage points on the implant surface to initiate proliferation and ensure mechanical stability. When the surface has a roughness much smaller than the cells' sizes, there may be an absence of adhesion sites. On the other hand, if the implant has a larger quantity of small peaks or valleys, the surfaces may be smooth and the cells, likewise, cannot be fixed [22].

Dental implant surfaces with Ra between 0.8 and 2.0 *μ*m have better osseointegration and mechanical stability than smooth or rougher surfaces. Implants with surface roughness between 0.8 and 1.5 *μ*m and with nanoroughness have the best success rates [23]. Possibly, the combination of micrometric roughness and the presence of graphene nanoparticles can improve dental implant osseointegration because cell adhesion on surfaces is directly related to chemical affinity and roughness. On dental implant titanium surfaces with irregularities larger than the size of the cell itself (osteoblast), there is lower adhesion. This happens due to the inability of the cell to establish a sufficient contact area with the substrate [24] Some cellular properties such as adhesion, morphological changes, proliferation, and differentiation are affected by the chemical composition, roughness, surface energy, and wettability of titanium [25]. On a smooth surface, polar groups increase wettability and nonpolar hydrophobic groups decrease it [26].

In vivo tests with rabbits showed that machined implants without surface treatment have Ra = 0.32 ± 0.03 *μ*m and a removal torque of 62.08 N·cm. Implants with Ra = 1.51 *μ*m are removed with a torque of 66.56 N·cm. The implant with greater Ra (1.0 *μ*m) needs a torque of 76.45 N·cm to be removed. These results show that osteoblasts have a better affinity for surfaces with medium roughness (Ra = 1.0 *μ*m) [20].

**Figure 8:**
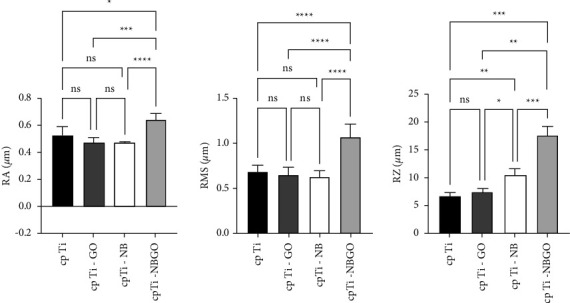
Tukey's statistical analysis of roughness parameters Ra, RMS, and Rz.

**Figure 9:**
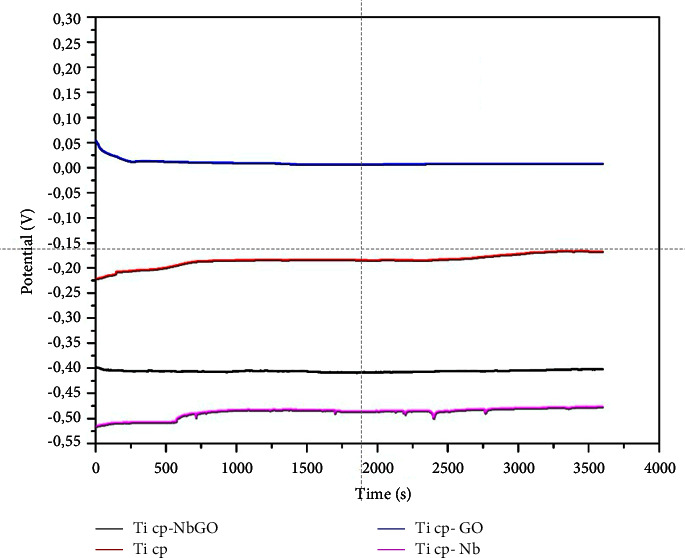
Open circuit potential measurement for cp Ti, cp Ti-GO, cp Ti-NB, and cpTi-NBGO in a physiological solution containing 0.9% NaCl.

Considering that RMS is the standard deviation of Ra, it can be said that the values of Ra and RMS are complementary.  Figure 8 shows the comparison of the RMS values among the groups. All groups showed a significant difference relative to the cp Ti-NBGO samples, and there is no significant difference among the other groups. To evaluate the coating adhesion and samples corrosion resistance, an open circuit potential (OCP) was monitored, and a polarization curve was obtained. The OCP is the equilibrium potential between the sample surface and the solution.  Figure 9 shows the OCP for cp Ti, cp Ti-NBGO, cp Ti-GO, and cp Ti-NB in a physiological solution containing 0.9% NaCl. It is observed that the potential of cp Ti is around −0.200 mV. When the samples were coated with GO, a displacement of the potential to positive values occurs, indicating that the corrosion increased. This indicative was also observed by Li et al. 2018 [27] in their analyses. The niobium oxide showed the opposite behavior since there is a shift to more negative values. This shift indicates a decrease in corrosion resistance.

**Figure 10:**
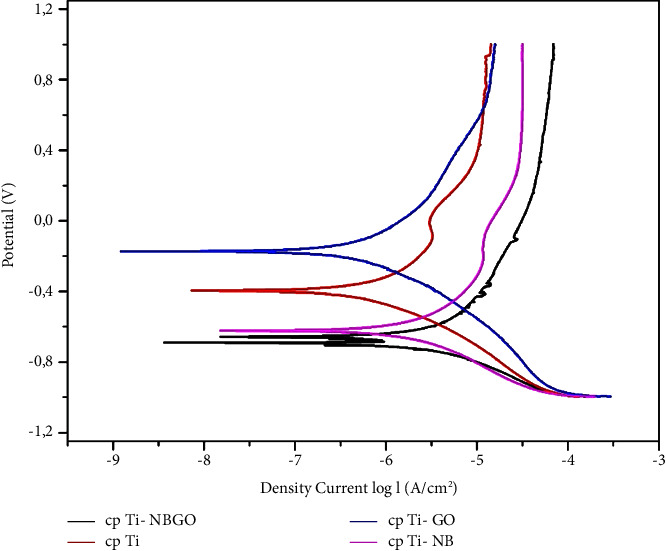
Polarization curve for cp Ti, cp Ti-GO, cpTi-NB, and cp Ti-NBGO in a physiological solution containing 0.9% NaCl.

Figure 10 shows the anodic potentiodynamic polarization curves for coated and uncoated samples.  Table 4 shows the values of open circuit potential, corrosion potential, and current density. It is observed that the two conditions with the presence of niobium were the ones that presented the highest value of current and corrosion potentials, which corroborates what was observed in the curves.

Figure 10 shows the polarization curves. All curves have a passive region. After niobium pentoxide deposition on the Ti surface, the current density increased in the anodic region, which means that the corrosion process increased. This behavior is due to the presence of a discontinuous film, which causes localized pits and increases the corrosion process. After graphene film coating, it is possible the corrosion resistance increases. This behavior may be associated with the formation of a protective layer that prevents the oxidation of titanium. This protection related to graphene film has been observed by different authors concerning other metallic alloys such as copper alloys and steels [27].

The deposition of graphene on metal surfaces forms a protective barrier that increases the corrosion resistance [27] and the dissolution process of different alloys. The graphene deposition efficiency against corrosion is on the order of 100 times higher than other processes. However, the deposition of large amounts of graphene induces galvanic corrosion [28]. The increased corrosion resistance and microbiological properties of GO are favorable for biomaterial applications. The film defects decrease the corrosion resistance and increase the ion release.

Table 4 shows the values of open circuit potential, corrosion potential, and current density. It is observed that the two conditions with the presence of niobium were the ones that presented the highest value of current and corrosion potentials, which corroborates with the potentiostatic curves. More negative OCP values are related to higher current values. Therefore, lower OCP values and higher current values are associated with lower corrosion resistance.

This decrease in corrosion resistance after the deposition of Nb_2_O_5_ may be associated with the lower stability of niobium oxide than titanium oxide, which is confirmed by the Ellingham diagram [29]. However, this Nb_2_O_5_ release is the desired effect since it may help osseointegration, increase in cell viability, and the proliferation of osteoblasts [30].

Based on the Ellingham diagram [28], it is possible to explain the decrease in corrosion resistance after the deposition of Nb_2_O_5_. This behavior may be associated with lower niobium oxide stability than titanium oxide. However, this Nb_2_O_5_ release is the desired effect since it may help osseointegration, increase in cell viability, and osteoblast proliferation [29].

## 5. Conclusion

Based on experimental results, it is possible to conclude that

- Wettability measurements showed that the Ti samples coated with graphene oxide (cp Ti-GO) had lower surface energy (12.94 ± 9.33 mJ/m^2^) than without coating cp Ti (51.61 ± 4.49 mJ/m^2^), cp Ti-NB (40.80 ± 13.47 mJ/m^2^), and cp Ti-NBGO (46.10 ± 6.38 mJ/m^2^). These surface energy values are adequate for protein and cell adhesion.
- Roughness measurements showed that uncoated samples had Ra = 0.52 ± 0.06 *μ*m, niobia coated samples had Ra = 0.44 ± 0.03 *μ*m, graphene oxide coated samples had Ra = 0.47 ± 0.04 *μ*m, and samples coated with both niobia and graphene oxide had Ra = 0.64 ± 0.47. Statistical analysis of the roughness parameters showed no statistically significant difference among samples from the first three groups and a significant difference with the last group.
- The anodic potentiodynamic polarization curves show that the graphene oxide coating increases corrosion resistance, the Nb_2_O_5_ coating decreases corrosion resistance, and the coating with both materials may be beneficial since the release of Nb_2_O_5_ helps in osseointegration, increasing cell viability, and proliferation of osteoblasts

## Figures and Tables

**Table 1:** Code used to identify the specimens.

Code	Surface treatment
cp Ti	cp Ti (control)
cp Ti-NB	cp Ti coated with Nb_2_O_5_
cp Ti-GO	cp Ti coated with GO
cp Ti-NBGO	cp Ti coated with Nb_2_O_5_ and GO

**Table 2:** Contact angle, surface energy, and statistical data.

Samples	Contact angle (^o^)			Surface energy (mJ/m^2^)		
	Mean ± Std deviation	*F*	*P*	Mean ± Std deviation	*F*	*P*
cp Ti	50.74 ± 4.6	26.6502	0.0002	51.61 ± 4.49	32.502	0.0017
cp Ti-NB	100.35 ± 10.85			40.80 ± 13.47		
cp Ti-GO	44.33 ± 10.0			12.94 ± 9.33		
cp Ti-NBGO	55.86 ± 6.1			46.10 ± 6.38		

**Table 3:** Mean and standard deviation (SD) of roughness parameters Ra, RMS, and Rz.

Samples	Ra (*μ*m)		RMS (*μ*m)		Rz (*μ*m)	
	Mean ± SD	*P* value	Mean ± SD	*P* value	Mean ± SD	*P* value
cp Ti	0.52 ± 0.06	*P* < 0.0001	0.67 ± 0.08	*P* < 0.0001	6.8 ± 0.60	*P* < 0.0001
cp Ti-Nb	0.44 ± 0.03		0.62 ± 0.06		10.60 ± 0.98	
cp Ti-GO	0.47 ± 0.04		0.6 ± 0.08		7.51 ± 0.71	
cp Ti-NbGO	0.64 ± 0.47		1.06 ± 0.14		17.64 ± 1.55	

**Table 4:** Open potential circuit (OCP), corrosion potential (*E*_corr_), and current density (*i*_corr_).

	OCP	*E *_corr_ (V)	*i *_corr_ (A/cm^2^)
cp Ti	−0.167 ± 0.01	−0.400 ± 0.02	5.46 × 10^−7^ ± 1.5 × 10^−7^
cp Ti-NB	−0.493 ± 0.04	−0.623 ± 0,05	1.48 × 10^−6^ ± 0.1 × 10^−6^
cp Ti-GO	0.007 ± 0.003	−0.160 ± 0.01	3.04 × 10^−7^ ± 0.2 × 10^−7^
cp Ti-NBGO	−0.643 ± 0.05	−0.667 ± 0.04	2.13 × 10^−6^ ± 0.1 × 10^−6^

## Data Availability

The data used to support the findings of this study are included in the article.
